# Recovery trajectories and predictors of symptom resolution in post-COVID-19 condition: a population-based cohort study

**DOI:** 10.1016/j.lanepe.2026.101802

**Published:** 2026-08-08

**Authors:** Jean-Patrick L. Brunet, Sonja L. van Ockenburg, Milad Leyli-Abadi, Gerton Lunter, Judith G.M. Rosmalen

**Affiliations:** a Department of Psychiatry, University of Groningen, University Medical Center Groningen, Groningen, the Netherlands; b Department of Endocrinology, University of Groningen, University Medical Center Groningen, Groningen, the Netherlands; c IRT SystemX, Palaiseau, France; d Department of Epidemiology, University of Groningen, University Medical Center Groningen, Groningen, the Netherlands; e Department of Internal Medicine, University of Groningen, University Medical Center Groningen, Groningen, the Netherlands

**Keywords:** Post-COVID-19 condition, Long COVID, Prognosis, Recovery, Population-based cohort, Longitudinal data

## Abstract

**Background:**

SARS-CoV-2 infection can lead to persistent symptoms, known as Post-COVID-19 Condition (PCC). Previous studies on PCC prognosis mainly looked at severe cases in rehabilitation settings and without knowledge about pre-infection symptom levels. Uncertainty remains about recovery trajectory in the general population, its predictors, and whether recovery differs by symptom.

**Methods:**

We analysed data from Lifelines, a prospective population-based observational cohort. Adults completed 31 COVID-19 questionnaires between March 2020 and October 2022 providing longitudinal symptom data to assess PCC status, and recovery. PCC was defined as at least one moderately severe symptom, among 12 identified as PCC-specific, that worsened 90–150 days after infection. Cox-proportional hazard models estimated recovery, defined as symptom decline to an individual’s pre-infection baseline, adjusted for symptoms present at PCC diagnosis, age, sex, BMI, smoking, hospitalization, vaccination, and comorbidities.

**Findings:**

We analysed time series of 809 cases (mean age 55.0; [SD 11.0]; 590 [73%] female; mean follow up 368 days; [SD 200]). Symptom decline to pre-infection baseline or below was observed in 558 participants; the Kaplan–Meier 24-month symptom decline estimate was 92%. Greater symptom burden was associated with a lower likelihood of recovery (HR per additional symptom 0.69, 95% CI 0.63–0.76), whereas younger age had a higher likelihood of recovery compared with middle adulthood (HR 1.52, 95% CI 1.06–2.18). Median time to symptom decline was 226 days (IQR 182–372), with most improvement within the first 235 days before slowing and plateauing.

**Interpretation:**

Most individuals with PCC recover, but older adults and those with multiple symptoms are at higher risk of prolonged illness, underscoring the need for ongoing support.

**Funding:**

This work was supported by The Netherlands Organisation for Health Research and Development (ZonMw) COVID-19 Program. The data collection in this project is co-financed by EU Horizon Europe Program grant Long Covid.

**Research in context**

**Evidence before this study**

We searched PubMed and the medRxiv repository for studies published between January 2020 and November 2025 in English, Dutch, or French that reported on the natural history, prognosis, or duration of Post–COVID-19 Condition (PCC), including studies using alternative terminology such as long COVID, post-COVID syndrome, or post-acute sequelae of SARS-CoV-2 infection. We identified two broad categories of studies: small cohort studies recruiting participants following COVID-19 hospital admission or those referred to post-COVID care pathways, and larger cohort studies established specifically to investigate PCC.

Many of these studies have important limitations. Definitions of PCC, recovery from PCC, and methodologies varied widely across studies. Earlier studies used heterogeneous time frames to define PCC, and studies show substantial heterogeneity in terms of symptom definitions, measurement instruments, and follow-up duration. Studies assessed between 18 and more than 60 symptoms, with follow-up ranging from three months to over two years, complicating comparisons. Many large cohort studies recruited participants through social media, direct contact, or patient organisations while smaller ones often focused on hospitalised or rehabilitation populations, raising concerns about selection bias. Most studies adjusted analyses for pre-existing medical conditions but did not adjust for individual pre-infection symptom levels, potentially biasing estimates of prevalence and recovery.

Across studies, trajectories of symptom burden of PCC cases over time varied widely. Some studies reported persistently high symptom burden in most cases with limited overall improvement, whereas others identified distinct subgroups characterised by rapid recovery (approximately 5–19%), sustained or intermittent symptoms with gradual improvement (35–91%), and little improvement or deterioration over time (4–46%). Severity of PCC and older age were consistently associated with poorer recovery, whereas other proposed risk factors appeared only in single studies or showed inconsistent associations.

**Added value of this study**

This study provides general population-based evidence on the recovery trajectory of PCC using a longitudinal cohort with detailed pre-infection and post-infection symptom data. By leveraging repeated symptom measurements before and after SARS-CoV-2 infection, and by comparing symptom levels with matched controls, this study improves the specificity of symptom attribution to PCC while accounting for pre-existing symptom burden. Unlike many earlier studies that focused on hospitalised or rehabilitation populations, this study uses an established cohort not originally designed for PCC research, which substantially reduces inclusion bias, and makes our conclusions more representative of the prognosis of PCC in the general population in the northern Netherlands from March 2020 to October 2022. Importantly, the dataset was collected in real time during the COVID-19 pandemic, and such comprehensive prospective data on post-viral sequelae remains relatively uncommon and can no longer be recreated.

Our findings show that most individuals with PCC return to at least their pre-infection symptom levels over time, but recovery within the population follows a non-linear trajectory characterised by a period of more rapid improvement followed by tapering and plateauing. A subgroup of PCC patients is characterized by slow or absent recovery over time. We found that the presence of multiple concurrent symptoms at the onset of PCC predicts a prolonged course and reduced chance of recovery. Contrary, onset of PCC in early adulthood appears to confer a higher likelihood for recovery. The results provide quantitative estimates of the timing of recovery and highlight heterogeneity in recovery trajectories that remains incompletely characterized in the PCC literature.

**Implications of all the available evidence**

Taken together with existing literature, these findings suggest that PCC is characterized by a heterogeneous presentation, with improvement possible for most affected individuals, but with diminishing likelihood of recovery as symptom load and duration increase. The identification of a critical period during which most recovery occurs has implications for clinical follow-up, patient counselling, and the timing of interventions. Accounting for pre-infection symptom levels is essential to avoid overestimation of persistent symptom burden and to better distinguish PCC from background symptom prevalence in the general population. By providing robust population-based estimates of recovery trajectories after SARS-CoV-2 infection, our findings equip public health authorities with the evidence needed to allocate resources, shape policies, and plan healthcare services that address the evolving burden of PCC.

## Introduction

SARS-CoV-2 infection can lead to persistent symptoms after recovery from the acute phase. This complex condition has been given many names such as Long COVID, Post acute COVID-19 syndrome, or Post-COVID-19 Condition (PCC). PCC incidence estimates vary, mostly ranging from 8% to 15% of SARS-CoV-2 infections.^1^, ^2^, ^3^ With more than 777 million COVID-19 cases reported worldwide by the WHO since the start of the pandemic and SARS-CoV-2 now being endemic, it appears that PCC will remain an important public health issue.^4^

For public health, not only the number of cases but also the rate of recovery is essential. While incidence estimates have improved over the pandemic, methodologically sound estimates of recovery rates are still largely lacking. Many studies used pre-selected small patient groups, often in a rehabilitation setting.^5^^,^^6^ Few studies exist on the prognosis of PCC in the general population. Results from those studies vary widely with some reporting consistent improvement over time while others show low or no improvement. Latent class mixed modelling in the French ComPaRe cohort identified three trajectories: no significant improvement (4% of patients), rapid improvement (5%), or sustained gradual improvement (91%) over two years, suggesting a relatively favourable prognosis of PCC.^7^ However, finite mixture modelling in the RECOVER-Adult US cohort over 12 months identified PCC trajectories characterized by improvement in 19%, highly persistent symptoms burden in 46%, and intermittent Long COVID-related symptoms for 35% of participants.^8^ The Long-COVID in Scotland Study (Long-CISS) analysed the entire infected group without defining PCC. This study showed that, beyond six months post-infection, there was little change in self-reported recovery or in the proportion of individuals experiencing at least one symptom linked to prior SARS-CoV-2 infection. At 12 months, 12% of people reported improvements, and 12% reported deterioration.^9^ A cross-sectional study using survival models on three French cohorts estimated the proportion of participants with at least one persistent symptoms at two months to be 32.5%, 18.4% at 6 months, 10.1% at 12 months, and 7.8% at 18 months.^10^ Showing continuous improvement over time with stronger recovery during the first year.^10^ A relatively consistent finding is that more severe PCC and older age are linked to lower or slower improvement over time.^7^, ^8^, ^9^, ^10^ However, findings on female sex, smoking, hospitalisation during acute infection, and comorbidities are inconsistent.^7^, ^8^, ^9^, ^10^

Those discrepancies likely reflect limitations in available studies, precluding reliable estimates of recovery rates.^7^, ^8^, ^9^, ^10^ First, participant recruitment often involved convenience sampling using (social) media, with the intent of studying PCC, potentially biasing towards including patients with more debilitating or longer lasting forms of the condition. Second, in the definition of PCC, previous studies on prognosis typically did not account for symptoms present prior to SARS-CoV-2 infection when defining cases. This increases the chance of falsely attributing symptoms to PCC, as most symptoms are nonspecific and widely present within the general population. Third, most of those studies used the total number of a wide range of symptoms as their primary outcome or did not verify the PCC specificity of symptoms. This ignores the fact that newly developed symptoms unrelated to PCC in the follow-up period might replace recovered PCC symptoms. Selection bias towards patients with more severe PCC, the inability to adjust for pre-existing symptoms, and reliance on the total number of symptoms without accounting for their specificity to PCC all tend to overestimate recovery times and may obscure a more favourable prognosis in the general population. Furthermore, Long-CISS suggests that recovery trajectories might differ between symptoms. Finally, most studies assessed symptom persistence infrequently, with common time frames being six, 12, 18, and 24 months post infection, which does not allow to observe the finer evolution of the condition over time. Given the above-mentioned limitations of previous studies, it is currently uncertain what the recovery trajectory of PCC looks like in the general population, what the predictors of recovery are, and if the recovery trajectory is different for different symptom types, once PCC has developed.

To address these questions, we analysed prognosis of PCC in Lifelines, a longitudinal population-based cohort with information on pre-infection symptom levels. As a general populations cohort that implemented a series of questionnaires during the COVID-19 pandemic, Lifelines provides both serial pre-infection and post-infection symptom data. We used these data to study recovery trajectories of PCC, adjusting for each individual’s pre-infection symptom level when defining both case status and recovery endpoints.

## Methods

### Study design and participants

This study is based on data collected within the Lifelines COVID-19 cohort study, an add-on study to the Lifelines cohort. Lifelines is a multi-disciplinary prospective population-based cohort study examining in a unique three-generation design the health and health-related behaviours of 167,729 persons living in the North of the Netherlands (ethnicity >98% white, 58% female). It employs a broad range of investigative procedures in assessing the biomedical, socio-demographic, behavioural, physical, and psychological factors which contribute to the health and disease of the general population, with a special focus on multi-morbidity and complex genetics.^11^ There were no specific inclusion criteria for the Lifelines study; exclusion criteria were severe mental illness, short life expectancy (<5 years) at time of inclusion, insufficient knowledge of the Dutch language to complete questionnaires, and not being able to visit a general practitioner.^11^ All Lifelines participants aged 18 years or older with a known email address received invitations to complete digital COVID-19 questionnaires (n = 144,145). Questionnaires were sent out from the end of March 2020 to mid-October 2022 with a frequency varying from weekly (until June 2020), bi-weekly (until August 2020), monthly (until June 2021) and finally every one to three months. We included data from 31 consecutive measurements, for which response rates varied between 49% and 28%.

The Lifelines cohort study and its add-on studies were approved by the Medical Ethical Committee of University Medical Center Groningen (2007/152) and conducted according to the principles of the Declaration of Helsinki; participants provided written informed consent.^11^

This study used the STrengthening the Reporting of OBservational studies in Epidemiology (STROBE) reporting guidelines for cohort studies.

### Procedures

The digital questionnaires contained questions on COVID-19 and somatic symptoms, which were used to assess COVID-19 status, PCC status, and PCC recovery status.

Participants were considered COVID-19 positive if they reported to have had a positive SARS-CoV-2 test or a physician’s diagnosis of COVID-19, based on the clinical case definition from the Dutch Institute for Public Health and the Environment. Physician diagnosis was included because SARS-CoV-2 testing was limited in the Netherlands until August 2020.

We used the WHO definition of PCC: persistent symptoms three months after the initial SARS-CoV-2 infection, with these symptoms lasting for at least two months with no other explanation.^12^

We assessed PCC using 12 symptoms, including ten core symptoms previously identified as marker of PCC in this cohort: chest pain, difficulties with breathing, pain when breathing, painful muscles, tingling extremities, lump in the throat, feeling hot and cold alternately, heavy arms or legs, general tiredness, ageusia or anosmia.^1^ Memory and concentration problems, added from questionnaire 21 onward, were additionally found to be increased 3–5 months after COVID-19. All 12 symptoms were thus validated as specific for PCC within this cohort. Symptom severity was self-reported on a 5-point Likert scale reflecting the degree to which participants were bothered by the symptom (1 = not at all, 5 = extremely) in the past seven days (until June 2020) or the past 14 days (from June 2020).

Baseline symptom levels were calculated by averaging each participant’s pre-infection symptom intensity scores per symptom, excluding the week before infection to avoid including acute disease symptoms. Participants without pre-infection data were excluded. Symptom persistence was operationalized by averaging questionnaire scores per symptom 90–150 days after infection. PCC positivity was defined as the presence of at least one persistent symptom with a mean intensity score equal or above three (moderate intensity or higher) that was increased by at least one point relative to the participant’s pre-infection baseline (Appendix, Supplementary Fig. S2). The threshold of moderate intensity was chosen to align with the WHO PCC definition, which requires symptoms to generally impact daily functioning. Comparing symptom levels to baseline helped address the criterion of absence of an alternative diagnosis.

SARS-CoV-2 variant was estimated based on date of infection and prevalence at the time: Wildtype prior to February 15th 2021, Alpha from February 15th 2021 to June 27th 2021, Delta from June 28th 2021 to December 26th 2021, and Omicron after December 27th 2021.^13^ Age was categorized in three groups for early adulthood, middle adulthood, and late adulthood (18–39, 40–59 and ≥60 years). Body mass index (BMI) (in kg/m^2^) was evaluated based on self-reported weight at time of inclusion in the Lifelines COVID-19 cohort and measured height during a previous Lifelines visit. BMI was subsequently categorized in normal, overweight and obesity (<25, ≥25 and <30, ≥30 kg/m^2^). No underweight category was considered due to the low number of participants with underweight. Smoking was assessed through the question “have you smoked in the previous 7/14 days?” with all participants reporting at least one positive answer during the study being categorized as smokers. Chronic diseases were assessed through single item questions assessing the presence of 17 diseases; a composite variable corresponding to the number of chronic diseases reported was constructed. From February 25th 2021 onward, vaccination status was assessed prior to infection through self-reporting. Participants with a full initial vaccination were categorized as being completely vaccinated. The questions on vaccination were added at the time the national vaccination campaign started in the Netherlands; participants with infection prior to this date were considered “not vaccinated”.^13^ Hospitalisation for a SARS-CoV-2 infection was assessed through self-report. Participants’ sex (male/female), age, and ethnicity was self-reported, with age defined at the time of entry in the Lifelines COVID-19 cohort study.

### Outcome and statistical analysis

The primary outcome was symptom decline, defined as the first questionnaire at which all symptoms contributing to the PCC categorization no longer met the definition (either at or below an individual’s pre-infection levels or <3). This was chosen to represent overall improvement, and individual symptom decline was not evaluated separately. To explore the influence of temporary fluctuation in symptom severity, we performed a sensitivity analysis with a more stringent definition requiring symptoms to not meet the definitions for two not necessarily consecutive questionnaires. In this case, the time of the first questionnaire was used as time of symptom decline.

Exploratory sensitivity analyses were conducted to evaluate the effect of missingness on the results. We performed multiple imputation using Multivariate Imputation by Chained Equations (MICE) for confounding variables with missing values (Comorbidities, Smoking, Hospitalisation and Vaccination).^14^ This model included all variables used in the Cox models with the addition of the number of questionnaires filled, follow-up duration, outcome indicator and Nelson-Aalen estimator as advised in the literature.^15^ Thirty datasets were imputed with a maximum of 20 iterations. Estimates were pooled using Rubin’s rules. To assess the robustness to the independent censoring assumption, two sensitivity analyses were conducted. Firstly, inverse probability of censoring weighting (IPCW) was applied using pooled logistic regression models for the censoring process across discrete follow-up intervals.^16^ Weighted Cox proportional hazards models were then fitted using robust variance estimation. Secondly, an imputation framework was used with varying censoring assumptions.^17^ Several censoring hypotheses were used to assess the robustness of the assumption of non-informative censoring.

Participant characteristics and missing data were reported as absolute numbers and percentages. Univariable analyses were performed using Kaplan–Meier estimators with comparison between strata using log-rank tests. Kaplan–Meier curves were displayed for the first 730 days for ease of representation, but the full length of available data was used in subsequent analyses. Multivariable analyses were performed using a Cox proportional hazard model on complete cases. For exploratory analysis, p-values were nominal and descriptive. All tests were two-sided. The proportional hazard assumption was verified by examining Schoenfeld residuals. SARS-CoV-2 variants violated the proportional hazards assumption and a model stratified by variant was used for the general analysis. A secondary Cox proportional hazards model was conducted to explore differences between symptoms. Predictors included symptoms present at the time of PCC definition, while the model was adjusted for age, sex, BMI, smoking, hospitalisation, vaccination status, and comorbidities, and was further stratified by variant. In total, 436 events were observed for 637 at risk participants with 20 variables in the regression models, sufficient for reliable regression estimates.^18^ Variables with sparse category specific events (<10) were retained in the multivariable model because they represented clinically relevant covariates and potential confounders. Sparse strata were interpreted cautiously because low event counts may yield unstable hazard ratio estimates. Analyses were performed using R version 4.4.2 with the survival, mice, and InformativeCensoring packages.^14^^,^^17^^,^^19^^,^^20^

### Role of the funding source

The funders of the study had no role in study design, data collection, data analysis, data interpretation, or writing of the report.

## Results

As in most studies involving repeated questionnaires, attrition and missing data will be unavoidable. In this study, several mechanisms lead to a seemingly high level of missingness (Appendix, Supplementary Fig. S1, Supplementary Table S2). All participants of the lifelines cohort with known email addresses were invited to participate (n = 140 145). From this population, 55% (n = 76 503) chose to participate in this study (mean age 53.6; SD 12.97; 61% female) and completed a total of 1,054,658 questionnaires (mean 13.8; SD 10.8). A known diagnosis of COVID-19 was an inclusion criterion for our study, leading to the inclusion of 13 191 patients in the study. 485 participants were structurally censored as baseline information could not have been collected due to study design (294 are left with lack of baseline information due to missingness). An additional 2946 participants were structurally censored due to a lack of information for PCC diagnosis (3364 were censored due to missingness). Overall, 3769 participants (28%) had to be censored due to missingness; 1048 (17%) of the remaining participants fulfilled the PCC definition. Of these, 239 lacked follow-up beyond 150 days post-infection and were excluded, with the majority, 128, being due to study design. The remaining 809 (mean age 55.0; SD 11.0; 73% female) had a maximum follow up of 920 days (mean 368; SD 200). No obvious baseline differences were observed between excluded and included groups (Appendix, Supplementary Table S1). Additionally, some degree of missingness was observed in confounding variables: chronic diseases (7%), smoking (<1%), hospitalisation during infection (1%) and vaccination status prior to infection (11%).

To evaluate potential participation bias, characteristics of participants and non-participants from the second Lifelines assessment wave were compared. Compared with non-participants, participants were older (mean age at second assessment 49.7 years [SD 12.8] versus 43.8 years [17.5]), more frequently female (60% versus 55%), and had a slightly higher BMI (26.0 kg/m^2^ [SD 4.3] versus 25.3 kg/m^2^ [4.9]).

**Fig. 1:**
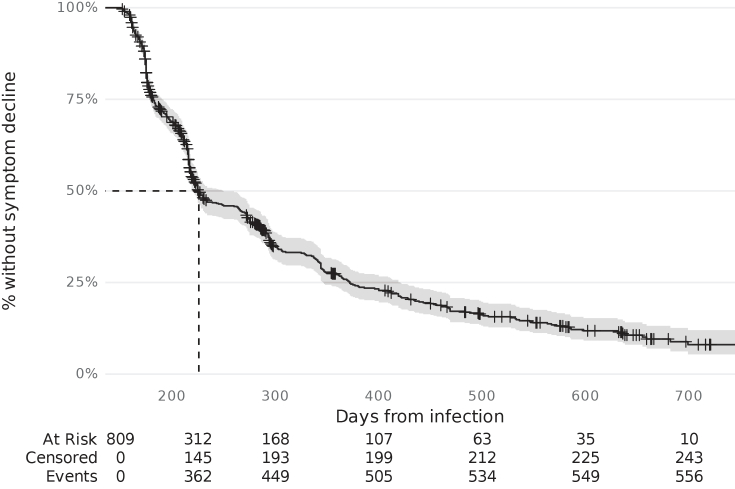
Symptom decline over time for all participants.

Symptom decline to a participant’s pre-infection baseline or below was observed in 558 (69%) participants fulfilling PCC criteria. The median time to this event was 226 days after infection (IQR 182–372). At six, 12, 18, and 24 months, the proportions of participants without symptom decline were 76.1% (standard error (SE) 1.5%), 26.1% (SE 1.9%), 14.6% (SE 1.6%), and 8.0% (SE 1.7%) respectively (Fig. 1). Visual inspection suggested a two-stage recovery profile, characterized by a rapid decline in symptoms during the first months after infection followed by slower recovery and an apparent plateau. To formally assess this pattern, we fitted piecewise exponential models with candidate changepoints between days 180 and 275 after infection at one-day intervals and selected the optimal model using the Akaike information criterion. This analysis supported a changepoint occurring at 235 days after infection, supporting a transition from a phase of faster recovery to a subsequent slower recovery phase. In the sensitivity analysis, using a more stringent definition for symptom decline, a similar profile was observed with a strong decline in the first 250 days followed by tapering until day 350, and finally a plateau (median 344 days CI 302–372) (Appendix, Supplementary Fig. S3). At six, 12, 18, and 24 months, the proportions of participants without symptom decline in the sensitivity analysis were 81.6% (SE 1.8%), 46.3% (SE 2.6%), 35.1% (SE 2.6%), and 34.5% (2.7%). However, formal changepoint analysis did not identify a distinct inflexion point in this sensitivity analysis, instead suggesting a gradual transition toward slower recovery rather than a discrete two-stage process.

**Fig. 2:**
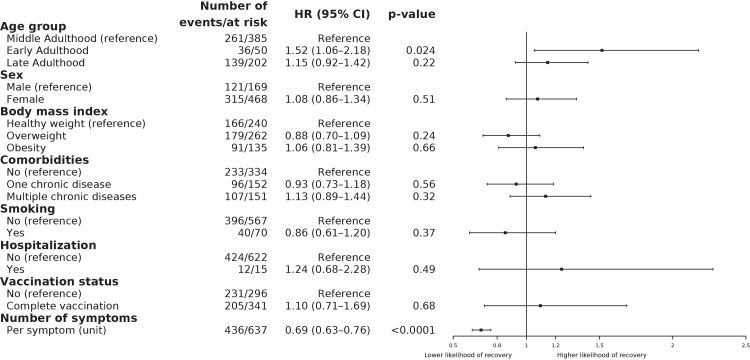
Forest plot of hazard ratios from multivariable Cox proportional hazards models stratified by SARS-CoV-2 variant.

Multivariable Cox models were used to identify predictors or recovery, including age, sex, BMI, comorbidities, smoking, hospitalisation, vaccination prior to infection, and number of symptoms; models were stratified by variant. Hazard Ratios are presented in Fig. 2. Early adulthood had a positive association with symptom decline when compared with middle adulthood (HR 1.52, 95% CI 1.06–2.18), while number of symptoms had a negative association (HR per additional symptom 0.69, 95% CI 0.63–0.76). Estimates for the remaining covariates were generally smaller and associated with wider confidence intervals that remained compatible with modest effects in either direction. Similar results were found in the sensitivity analysis, using a more stringent definition for symptom decline (Appendix, Supplementary Fig. S4). Because severe acute COVID-19 and hospitalisation were relatively uncommon in this cohort, caution is warranted when extrapolating these findings to patients with severe acute infection.

**Fig. 3:**
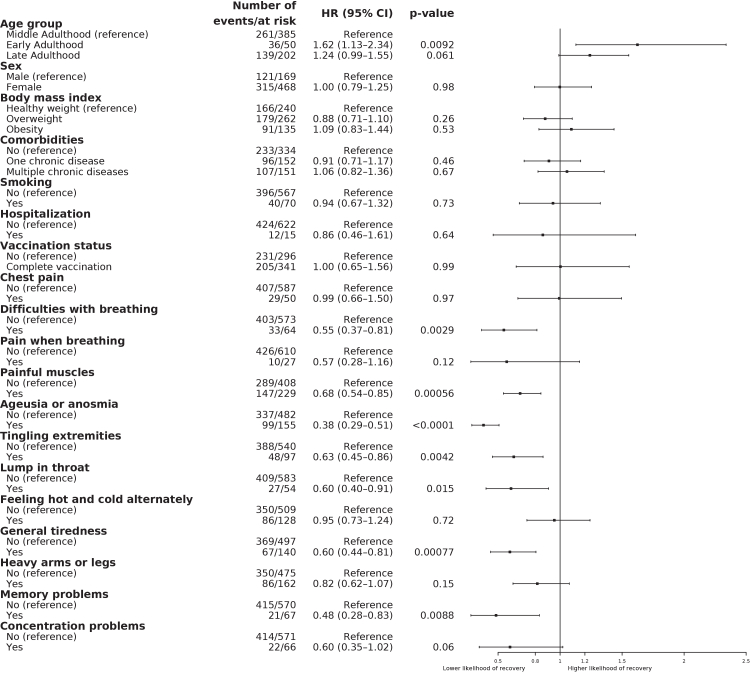
Forest plot of hazard ratios from multivariable Cox proportional hazards models including symptoms stratified by SARS-CoV-2 variant.

A second multivariable Cox regression model examined effects of symptom type on recovery trajectory, adjusted for previous covariates and stratified by variant (Fig. 3). Difficulties with breathing, painful muscles, ageusia or anosmia, tingling extremities, lump in throat, heavy arms or legs, general tiredness and memory problems were all significantly negatively associated with symptom decline. Comparable patterns were identified in the sensitivity analyses, although not all associations were significant (Appendix, Supplementary Fig. S5, 7, and S9).

Multiple imputation of the confounding variables was conducted as a sensitivity analysis to assess the robustness of the findings. The protective effect of early adulthood was found to be no longer statistically significant while number of symptoms remained significantly associated with poorer recovery outcomes (Appendix, Supplementary Fig. S6).

The sensitivity analysis conducted to test the robustness of the finding to informative censoring consistently found, across multiple censoring assumptions and models, that number of symptoms at diagnosis was a statistically significant negative predictor of recovery (Appendix, Supplementary Fig. S8). Other factors were not found to be statistically significant.

## Discussion

This is the first study able to investigate recovery in PCC patients identified in a general population by looking at symptom decline to pre-infection levels. Our data help clarify that prognosis may differ across the broad spectrum of PCC severity, with a polysymptomatic subgroup experiencing slow or absent recovery and others achieving favourable long-term outcomes. While symptom decline was observed in most participants (Kaplan–Meier estimate, 92%), it was a fairly long process with a median time to return to pre-infection symptom levels of 226 days. Symptom decline presented a two-stage profile, with most participants improving in the first 250 days followed by tapering, and a plateau. Thus, recovery becomes less likely for patients whose condition has become more chronic. The presence of multiple PCC symptoms predicted a prolonged course of PCC, whereas younger age was associated with a higher chance of recovery. Symptoms associated with prolonged recovery include those that directly impair functioning, such as cognitive problems, and general fatigue. Our findings demonstrate that the extended recovery time among individuals with PCC further underscores its considerable public health significance, especially given its high prevalence.

This study has several unique strengths. As a prospective cohort recruited before reports of PCC, inclusion bias is limited. Whereas clinical PCC cohorts are likely biased towards more chronic and severe PCC, our cohort identified cases within the same post-infection window and represents the full spectrum from mild to severe. Our recovery estimates are thus likely more accurate than those of previous studies that recruited participants who already obtained a PCC diagnosis. Additionally, continuous sampling during the pandemic is unique in its duration (up to 920 days) and frequency (31 assessment points) with most other studies limited to a couple of observations post-infection. This design has major advantages for both classification of PCC, and definition of recovery status. It allowed us to assess PCC status based on newly developed or substantially increased symptoms after COVID-19, compared to an individual’s pre-infection levels for symptoms assessed to be specific of PCC. This uniquely addresses the “not otherwise explained” part of PCC definition, and to operationalize recovery as return to an individual’s baseline symptom level as assessed pre-infection, limiting recall biases. To our knowledge this is the only dataset allowing for this type of characterization, with most other studies relying on post-infection observations and self-reported PCC status.

Several limitations should be considered. Questionnaire intervals varied and became longer during the pandemic, possibly delaying observation of symptom decline and extending recovery times. Since PCC symptoms often fluctuate, assessment at single time points could overestimate recovery. A sensitivity analysis requiring two questionnaires not meeting the PCC definition resulted in longer recovery trajectories, but similar patterns and predictors.

Cognitive symptoms were assessed later in the study, resulting in shorter follow-up, but their association with symptom decline was comparable to other symptoms, suggesting limited impact on recovery time. Treatment data were unavailable, but the limited availability of evidence-based PCC treatments in the Netherlands during the study period makes substantial confounding unlikely. Reinfections and intercurrent illnesses could not be reliably accounted for, particularly following changes in national testing policies, potentially affecting recovery estimates.

While studies lacking pre-infection symptom data may overestimate PCC cases, our stricter case definition (increased symptoms 3–5 months after infection) may have underestimated some cases, particularly those with symptoms resolving shortly after 3 months post-infection. This approach ensured consistency with previous Lifelines research but may have biased the sample toward more persistent cases.

As the cohort was initiated before PCC was formally recognised, post-exertional malaise and brain fog were not assessed, and mental health symptoms were not included. Consequently, PCC occurrence may have been underestimated, and prognosis could not be evaluated in patients primarily presenting with these symptoms. Therefore, the reported recovery trajectories may not fully reflect the broader experience of PCC.

While pre-infection symptom data and symptom-specific assessments reduced misclassification due to pre-existing conditions or alternative diagnoses, these cannot be fully excluded. Some COVID-19 diagnoses were self-reported, which may also have led to misclassification despite additional verification questions.

Attrition and missing data may have introduced bias. Some exclusions were inherent to the study design (e.g. early or late infections with insufficient data) and retention may have been influenced by PCC severity, introducing possible informative censoring. Although baseline characteristics of included and excluded PCC groups were similar, residual biases cannot be ruled out.

Cohort participants were slightly older, more often female, and had a slightly higher BMI than non-participants, suggesting some selection bias. Restricting prognostic analyses to PCC cases, may have caused index-event and collider bias. Sensitivity analyses using IPCW, multiple imputation, and gamma-based approaches supported the main findings, especially for PCC severity, while age associations were less consistent and should be interpreted with caution.

Most patients with PCC experienced symptom improvement within the first 250 days; however, a substantial subset experiences persistent symptoms beyond this period without returning to pre-infection levels. The definition of symptom decline used in this study does not fully equate to recovery, as demonstrated by the sensitivity analysis, which shows a higher number of patients not recovered two years after infection (35% versus 8%). Attribution of persistent symptoms to the initial infection also becomes increasingly uncertain over time, as several symptoms commonly reported in PCC are also common in the general population. This uncertainty was partly mitigated by restricting recovery analyses to symptoms present at PCC onset and excluding symptoms that emerged during follow-up.

The long recovery trajectories observed in our study are in line with previous reports showing limited improvement for patients with long standing PCC.^6^, ^7^, ^8^, ^9^ In a convenience sample of 2197 patients with PCC, 90.8% showed only a slow decline in symptom occurrence over 2 years, while participants with a high number of symptoms (4.9%) were unlikely to improve.^7^ Similarly, more than half of 806 patients referred to a PCC clinic showed no improvement after 1.5 years.^6^ An analysis combining three French cohorts also found that most recovery occurred within the first 6 months and subsequently slowed.^10^ Most studies did not report the fast decline observed during the first 250 days in our study.^10^

Differences in study populations, case definitions, and observation periods may explain this discrepancy. Most PCC studies use cohorts that are case-based rather than population-based and are therefore more likely to include participants with long-term or debilitating forms. This is particularly true for previous studies recruiting through social media campaigns, patient organizations, or dedicated PCC clinics.^6^^,^^7^ The long-COVID in Scotland Study used a general population cohort with matched controls, but did not adjust for pre-infection symptoms, and reported high prevalence of symptoms in the non-infected group.^9^ Similar recovery trajectories were reported in another population-based cohort study combining repeated observations within an existing cohort.^10^ Our cohort was established before PCC was recognised and followed participants from the beginning of the pandemic, likely reducing selection towards more severe or chronic cases. Moreover, PCC was defined within a specified post-infection period using symptoms associated with COVID-19, and pre-infection data allowed us to distinguish new or substantially worsened symptoms from pre-existing complaints.

The study period from March 2020 to October 2022, also limits generalizability as most participants were unvaccinated, and infected by pre-Omicron variants. Recovery may differ among vaccinated individual infected with Omicron. Contextual difference may further contribute to differences between cohorts. In the Netherlands, the northern provinces in the early stages of the pandemic conducted testing and confinement while the rest of the country favoured herd immunity.^21^ Although these policies might have had limited effects on the clinical course of established PCC, associated differences in social isolation, healthcare access, and other contextual factors may have influenced recovery.

Differences between study populations might also explain the differences in predictors of recovery. Our study is based on data collected between March 2020 to October 2022 in a sample representative of the northern Netherlands, and as such might not represent the full picture of PCC phenotypes. In accordance with the literature, a higher PCC symptom burden was associated with a prolonged course of PCC, with each additional symptom reducing the likelihood of recovery (HR 0.69, 95% CI 0.63–0.76), whereas younger age was associated with a greater likelihood of recovery (HR 1.52, 95% CI 1.06–2.18 for early versus middle adulthood).^7^, ^8^, ^9^ We also confirmed earlier findings on the absence of an association for BMI, comorbidity, and vaccination status and found no association with smoking. We analysed chronic conditions as a composite variable corresponding to the number of comorbidities. As a result, direct effects of specific chronic conditions could not be identified. Previous findings on sex and hospitalisation during acute infection were mixed; we did not find associations with PCC recovery status. Another Lifelines study found that female sex, higher BMI, comorbidities, being unvaccinated, and hospitalisation were associated with developing PCC, while age and smoking did not affect the risk.^22^ This suggests that risk factors for developing PCC might differ from those associated with recovery.^15^

While previous studies have suggested that virus variant is important for the risk to develop PCC, data on its association with recovery are lacking.^23^^,^^24^ Assessing differences in symptom decline between virus variants is challenging due to several factors. Classification of SARS-CoV-2 variants was based on the predominant circulating variant during the infection period rather than on viral genotyping, which may have introduced some misclassification. Varying observation periods were used, ranging from 900 days for Wildtype infections versus only 300 days for Omicron infections. Additionally, questionnaire intervals increased throughout the study. While Omicron was associated with a more rapid initial symptom decline, limited follow-up prevented longer-term conclusions. No significant differences in symptom decline were observed between the other variants. Those conclusions may be limited as additional temporal effects may have been present and indirectly measured through the variants which were defined as specific time intervals. For instance, vaccination access, load on the health care system, awareness of PCC and associated care varied over time and could have confounded the observed variant effects.^25^

This study provides a rare and comprehensive description of the prognosis of PCC within a general population-based cohort. Our findings suggest that the broadly inclusive WHO definition identifies both mild monosymptomatic forms with a long-term favourable prognosis as well as a clinically important subgroup with much slower recovery. While biological heterogeneity likely plays an important role in symptom persistence, differences between symptoms may also reflect variation in symptom measurement properties or background prevalence. Symptom improvement becomes less likely after approximately 235 days and individuals with more than two persistent symptoms are at elevated risk of long-term impairment, underscoring the need for sustained care pathways and follow-up strategies for this group. Younger age was found to be associated with earlier symptom improvement; however, this concerned a minority of overall cases. The information provided by this study is essential for planning clinical services and informing public-health strategies.

Importantly, these long-term outcomes are likely relevant beyond COVID-19, as persistent post-infectious symptoms are known to occur after many infections and likely share underlying mechanisms. What was unique is not SARS-CoV-2 itself, but the pandemic circumstances which exposed the general population to a novel virus. This provided an extraordinary opportunity that is rarely available for other infections: to prospectively study post-infectious sequelae at scale. It should be noted that these findings were shaped by unique pandemic-related circumstances, including social disruption, heightened disease awareness, changes in healthcare access, and the introduction of vaccination, all of which may have influenced symptom reporting and recovery trajectories. This context may limit the generalizability of our results to current COVID-19 cases or other post-infectious conditions. Finally, the analyses focused on time to first recovery event and therefore did not specifically model symptom fluctuation, temporary remission, or recurrence over time, which may require alternative approaches such as multistate modelling. While such large-scale studies may not be feasible now that the pandemic has ended, future research comparing recovery trajectories and predictors across smaller cohorts affected by different infections would further advance our understanding of persistent post-infectious symptoms.

## Contributors

JLB analysed the data, conceptualised the analyses, and wrote the first version of the manuscript. SLvO, MLA, GL and JGMR helped with conceptualising the analyses, interpreting the results, and critically revised the manuscript. JLB, MLA and SLvO accessed and verified the reported underlying data. JGMR conceived the study’s design, helped conceptualise the analyses, interpreted the results, and critically revised the manuscript.

## Data sharing statement

Data may be obtained from a third party and are not publicly available. Researchers can apply to use the Lifelines data used in this study. More information about how to request Lifelines data and the conditions of use can be found on their website (https://www.lifelines-biobank.com/researchers/working-with-us).

## Declaration of interests

ML reports institutional internal support from IRT SystemX related to the submitted work, including allocated work time, payment of workshop organisation costs, and reimbursement of travel costs for collaboration with University Medical Center Groningen (UMCG). ML also reports support from UMCG for access to Lifelines data. ML reports a leadership role on behalf of IRT SystemX in the AI4REALNET European project, funded by the European Commission, focusing on AI–human interaction paid to institution. No direct personal payments were received.

SLvO reports serving, as a member of the ME/CFS guideline committee on behalf of the Dutch Internist Society (NIV), paid to the institution.

JGMR reports research grants from the Netherlands Organisation for Health Research and Development (ZonMw; grant numbers 10430302110002 and 11080022410018), paid to her institution, in support of the submitted work. JGMR also reports research grants from the European Union, the National Institute of Mental Health, and the Netherlands Organisation for Scientific Research (NWO; Vici grant Vi.C.191.021), paid to her institution, outside the submitted work. JGMR reports royalties from Lannoo Publishers for a handbook on persistent somatic symptoms, paid to her institution. JGMR reports consulting fees from the Knowledge Institute of Medical Specialists for membership of the Somatic Symptom Disorder guideline committee, and from the Dutch Health Council for membership of the committee preparing advice on post-COVID, both paid to her institution. JGMR reports receiving the Wayne Katon Research Award from the Academy of Consultation-Liaison Psychiatry and the J.J. Groen Senior Award 2024, both paid to her institution. JGMR also reports support from National Taiwan University Hospital Yunlin Branch to cover travel costs for an invited lecture. JGMR serves, unpaid, as a member of the Data Safety Monitoring Board and Advisory Board of the SOMACROSS research unit (FOR 5211), as vice-president of the European Association of Psychosomatic Medicine, and as president of the Dutch Network Persistent Somatic Symptoms (NALK).

All other authors declare no competing interests.

## References

[1] Ballering A.V., van Zon S.K.R., Olde Hartman T.C., Rosmalen J.G.M. (2022). Persistence of somatic symptoms after COVID-19 in the Netherlands: an observational cohort study. Lancet.

[2] Hastie C.E., Lowe D.J., McAuley A. (2023). True prevalence of long-COVID in a nationwide, population cohort study. Nat Commun.

[3] Ayoubkhani D., Bosworth M.L., King S. (2022). Risk of long COVID in people infected with severe acute respiratory syndrome Coronavirus 2 after 2 doses of a Coronavirus disease 2019 vaccine: community-based, matched cohort study. Open Forum Infect Dis.

[4] COVID-19 cases | WHO COVID-19 dashboard. https://data.who.int/dashboards/covid19/cases.

[5] Liira H., Garner P., Malmivaara A. (2024). Prognosis of patients with post-Covid-19 condition: prospective cohort cluster analysis at one year. J Psychosom Res.

[6] Agergaard J., Gunst J.D., Schiøttz-Christensen B., Østergaard L., Wejse C. (2023). Long-term prognosis at 1.5 years after infection with wild-type strain of SARS-CoV-2 and Alpha, Delta, as well as Omicron variants. Int J Infect Dis.

[7] Servier C., Porcher R., Pane I., Ravaud P., Tran V.T. (2023). Trajectories of the evolution of post-COVID-19 condition, up to two years after symptoms onset. Int J Infect Dis.

[8] Thaweethai T., Donohue S.E., Martin J.N. (2025). Long COVID trajectories in the prospectively followed RECOVER-adult US cohort. Nat Commun.

[9] Hastie C.E., Lowe D.J., McAuley A. (2023). Natural history of long-COVID in a nationwide, population cohort study. Nat Commun.

[10] Robineau O., Zins M., Touvier M. (2022). Long-lasting symptoms after an acute COVID-19 infection and factors associated with their resolution. JAMA Netw Open.

[11] Sijtsma A., Rienks J., Van Der Harst P., Navis G., Rosmalen J.G.M., Dotinga A. (2022). Cohort profile update: Lifelines, a three-generation cohort study and biobank. Int J Epidemiol.

[12] Soriano J.B., Murthy S., Marshall J.C., Relan P., Diaz J.V. (2022). A clinical case definition of post-COVID-19 condition by a Delphi consensus. Lancet Infect Dis.

[13] Corona actueel | RIVM. https://www.rivm.nl/corona/actueel.

[14] van Buuren S., Groothuis-Oudshoorn K. (2011). mice: multivariate imputation by chained equations in R. J Stat Softw.

[15] White I.R., Royston P. (2009). Imputing missing covariate values for the cox model. Stat Med.

[16] Robins J.M., Hernán M.Á., Brumback B. (2000). Marginal structural models and causal inference in epidemiology. Epidemiology.

[17] Jackson D., White I.R., Seaman S., Evans H., Baisley K., Carpenter J. (2014). Relaxing the independent censoring assumption in the Cox proportional hazards model using multiple imputation. Stat Med.

[18] Peduzzi P., Concato J., Feinstein A.R., Holford T.R. (1995). Importance of events per independent variable in proportional hazards regression analysis II. Accuracy and precision of regression estimates. J Clin Epidemiol.

[19] R Core Team (2024).

[20] Therneau T.M., Grambsch P.M. (2000).

[21] Hoekman L.M., Smits M.M.V., Koolman X. (2020). The Dutch COVID-19 approach: regional differences in a small country. Health Policy Technol.

[22] van Zon S.K.R., Ballering A.V., Brouwer S., Rosmalen J.G.M. (2023). Symptom profiles and their risk factors in patients with post-COVID-19 condition: a Dutch longitudinal cohort study. Eur J Public Health.

[23] Antonelli M., Pujol J.C., Spector T.D., Ourselin S., Steves C.J. (2022). Risk of long COVID associated with delta versus omicron variants of SARS-CoV-2. Lancet.

[24] Lok L.S.C., Sarkar S., Lam C.C.I. (2024). Long COVID across SARS-CoV-2 variants: clinical features, pathogenesis, and future directions. MedComm Futur Med.

[25] Pastorello A., Meyer L., Coste J. (2025). Temporal changes in the risk of six-month post-COVID symptoms: a national population-based cohort study. Am J Epidemiol.

