# Supplementary material for: Recovery trajectories and predictors of symptom resolution in post-COVID-19 condition: a population-based cohort study
Source: Lancet Reg Health Eur. 2026 Aug 8;69:101802. doi: 10.1016/j.lanepe.2026.101802 (PMC13476563; doi:10.1016/j.lanepe.2026.101802)
Supplement: Supplementary file 1 [file mmc1.pdf]

Supplements

Recovery trajectories and predictors of symptom resolution in post-COVID-19 condition: a population-based cohort study

Content

Figure S1 - Study inclusion profile	P2
Table S1 - Baseline characteristics	P3
Figure S2 - Representation of PCC diagnostic window. Light grey represents the window of observation of pre-infection value calculation; dark grey corresponds to the window of observation for PCC diagnosis.	P4
Figure S3 - Sensitivity analysis using a stricter definition of symptom recovery based on two consecutive questionnaires	P5
Figure S4 - Sensitivity analysis: - Forest plot of hazard ratios from multivariable Cox proportional hazards models stratified by SARS-CoV-2 variant using the two-questionnaire definition of return to baseline symptom level. Category with less than 10 are reported as <10 to preserve participants anonymity.	P5
Figure S5 - Sensitivity analysis: - Forest plot of hazard ratios from multivariable Cox proportional hazards models for PCC symptoms stratified by variant considering return to baseline level for two questionnaires. Category with less than 10 are reported as <10 to preserve participants anonymity.	P6
Figure S6 - Forest plot of hazard ratios from multivariable Cox proportional hazards models stratified by SARS-CoV-2 variant following multiple imputation.	P7
Figure S7 - Forest plot of hazard ratios from multivariable Cox proportional hazards models including symptoms stratified by SARS-CoV-2 variant following multiple imputation.	P8
Figure S8 - Sensitivity analyses of adjusted Cox proportional hazards models for recovery. Hazard ratios (HRs) with 95% confidence intervals are presented for the primary complete-case model, multiple imputation, IPCW after imputation for informative censoring, and gamma sensitivity analyses in the imputed dataset. HRs <1 indicate lower recovery likelihood and HRs >1 indicate higher recovery likelihood.	P9
Figure S9 - Sensitivity analyses of adjusted Cox proportional hazards models including symptoms for recovery. Hazard ratios (HRs) with 95% confidence intervals are presented for the primary complete-case model, multiple imputation, IPCW for informative censoring, and gamma sensitivity analyses. HRs <1 indicate lower recovery likelihood and HRs >1 indicate higher recovery likelihood.	P10
Table S2 Questionnaire participation and number of observations contributing to baseline and PCC symptom estimation.	P11

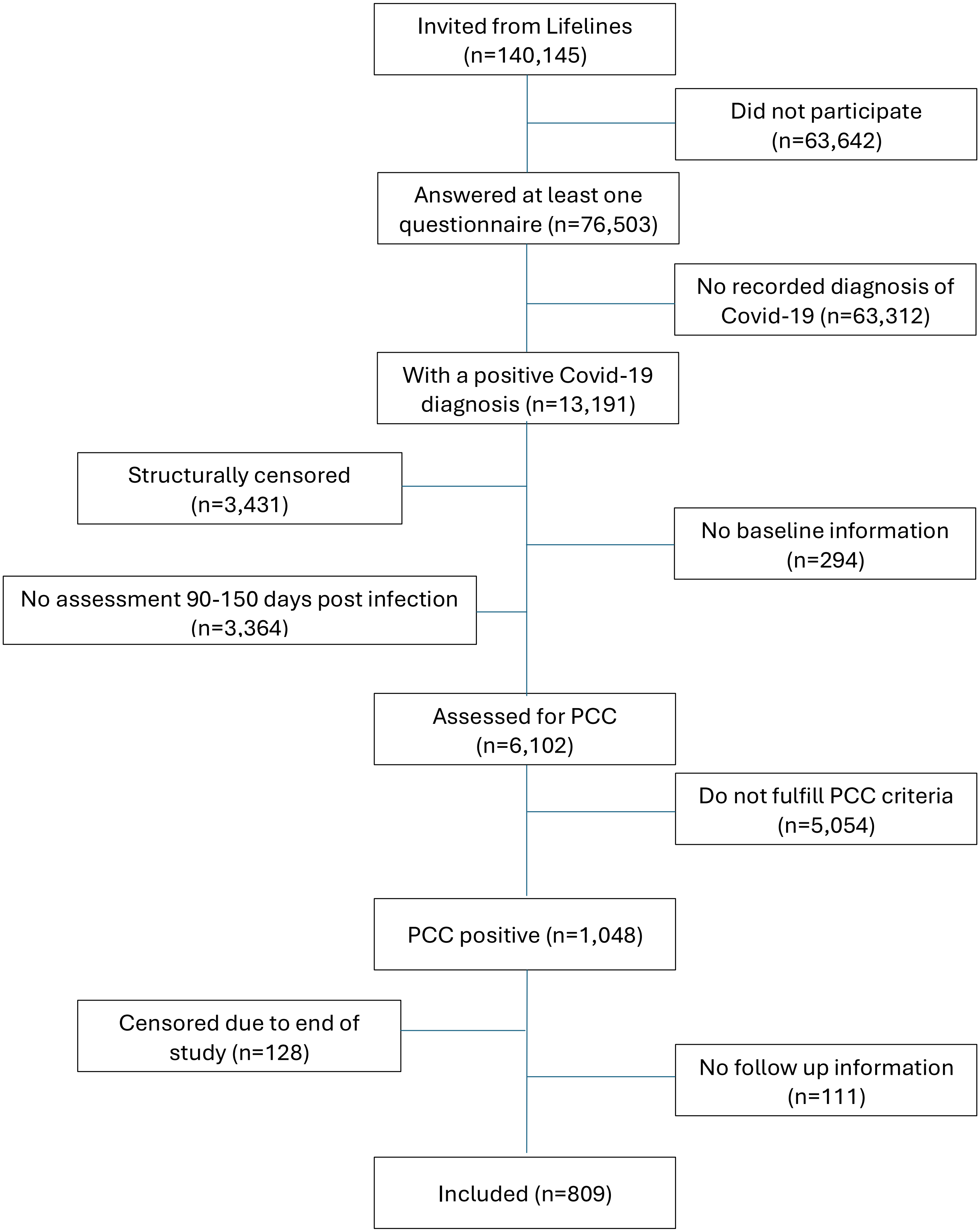

Figure S1 - Study Inclusion profile

Table S1 - Baseline characteristics

	SARS-CoV-2 positive (n=13,191)	SARS-CoV-2 positive With assessment prior and 90-150 days post infection (n=6,102)	PCC Positive (n=1,048)	PCC Negative (n=5,054)	PCC Positive with follow up (n=809)	PCC Positive without follow up (n=239)
**Completed questionnaires**	21·4 (8·9)	25·7 (6·4)	24·2 (7·0)	26·0 (6·2)	25·3 (6·5)	20·6 (7·5)
**Age (years)**						
18-39	1520 (12%)	558 (9%)	98 (9%)	460 (9%)	68 (8%)	30 (13%)
40-59	7006 (53%)	3077 (50%)	628 (60%)	2449 (48%)	491 (61%)	137 (57%)
≥60	4665 (35%)	2467 (40%)	322 (31%)	2145 (42%)	250 (31%)	72 (30%)
**Sex**						
Male	4631 (35%)	2196 (36%)	287 (27%)	1909 (38%)	219 (27%)	68 (28%)
Female	8560 (65%)	3906 (64%)	761 (73%)	3145 (62%)	590 (73%)	171 (72%)
**BMI (kg/m^2)**						
<25	5830 (44%)	2700 (44%)	409 (39%)	2291 (45%)	305 (38%)	104 (44%)
≥25 and <30	5173 (39%)	2408 (39%)	415 (40%)	1993 (39%)	325 (40%)	90 (38%)
≥30	2188 (17%)	994 (16%)	224 (21%)	770 (15%)	179 (22%)	45 (19%)
**Chronic disease**						
No	7948 (60%)	3824 (63%)	517 (49%)	3307 (65%)	404 (50%)	113 (47%)
One	2212 (17%)	1107 (18%)	231 (22%)	876 (17%)	171 (21%)	60 (25%)
Multiple	1643 (12%)	796 (13%)	219 (21%)	577 (11%)	174 (22%)	45 (19%)
Unknown	1388 (11%)	375 (6%)	81 (8%)	294 (6%)	60 (7%)	21 (9%)
**Smoking***						
Yes	- (10%)	- (10%)	- (11%)	- (9%)	- (11%)	- (11%)
No	- (89%)	- (90%)	- (89%)	- (91%)	- (89%)	- (89%)
Unknown	- (1%)	- (0%)	- (0%)	- (0%)	- (0%)	- (0%)
**Hospitalization***						
Yes	190 (1%)	88 (1%)	37 (4%)	51 (1%)	- (4%)	- (2%)
No	12663 (96%)	5892 (97%)	989 (94%)	4903 (97%)	- (95%)	- (93%)
Unknown	338 (3%)	122 (2%)	22 (2%)	100 (2%)	- (1%)	- (5%)
**Vaccination prior to infection**						
Full	6701 (51%)	3524 (58%)	526 (50%)	2998 (59%)	370 (46%)	156 (65%)
Partial	562 (4%)	193 (3%)	34 (3%)	159 (3%)	24 (3%)	10 (4%)
No	4492 (34%)	1969 (32%)	378 (36%)	1591 (31%)	326 (40%)	52 (22%)
Unknown	1436 (11%)	416 (7%)	110 (10%)	306 (6%)	89 (11%)	21 (9%)
**Variant**						
Wildtype	2747 (21%)	1368 (22%)	253 (24%)	1115 (22%)	236 (29%)	17 (7%)
Alpha	1417 (11%)	535 (9%)	131 (13%)	404 (8%)	118 (15%)	13 (5%)
Delta	1096 (8%)	594 (10%)	129 (12%)	465 (9%)	119 (15%)	10 (4%)
Omicron	7931 (60%)	3605 (59%)	535 (51%)	3070 (61%)	336 (42%)	199 (83%)
**Number of symptoms at diagnosis**						
1	n.a.	n.a.	621 (59%)	n.a.	471 (58%)	150 (63%)
2	n.a.	n.a.	196 (19%)	n.a.	163 (20%)	33 (14%)
3	n.a.	n.a.	96 (9%)	n.a.	72 (9%)	24 (10%)
>3	n.a.	n.a.	135 (13%)	n.a.	103 (13%)	32 (13%)

Data are n (%), mean (SD). BMI= body-mass-index. *When a category contains less than 10 participants, only percentages are reported to preserve anonymity.

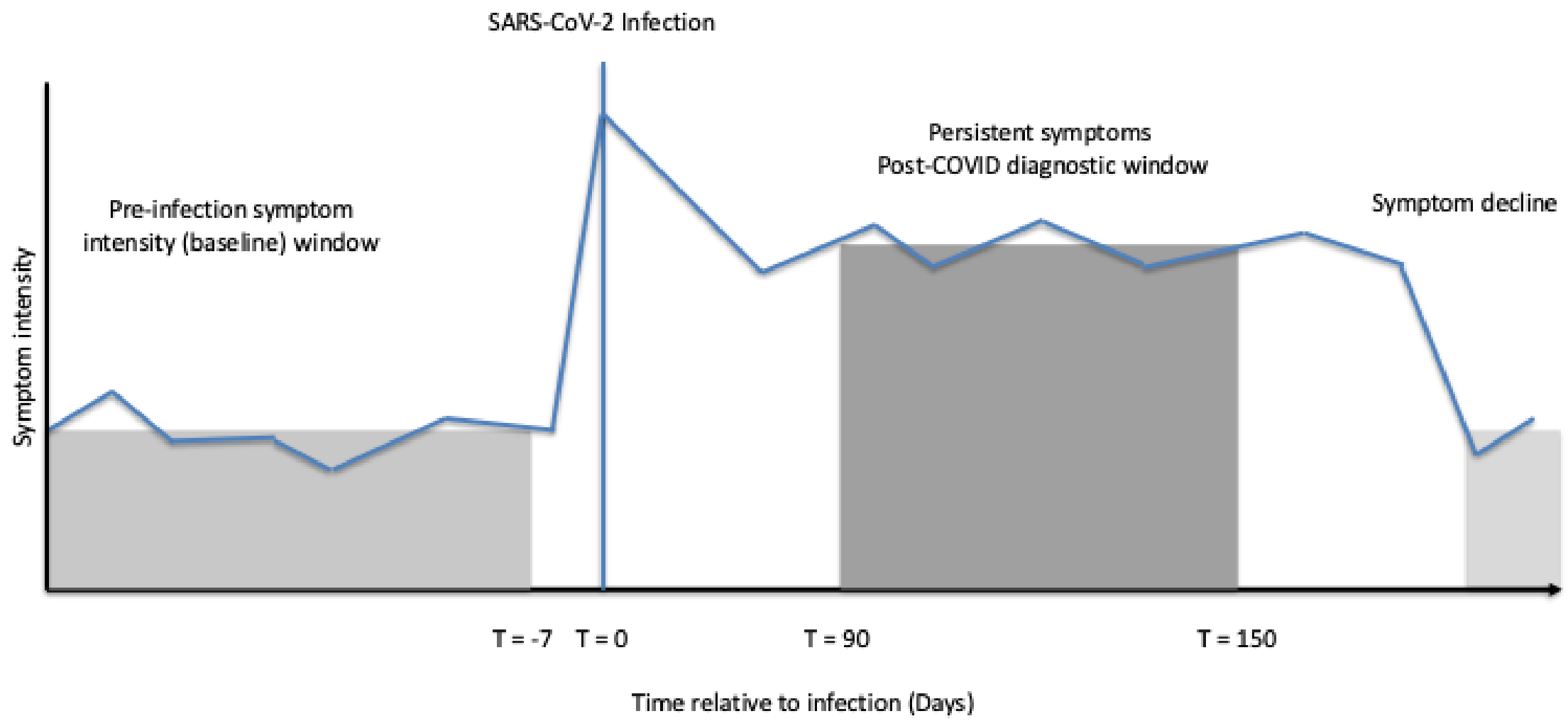

Figure S2 - Representation of PCC diagnostic window. Light grey represents the window of observation of pre-infection value calculation; dark grey corresponds to the window of observation for PCC diagnosis.

Sensitivity analysis

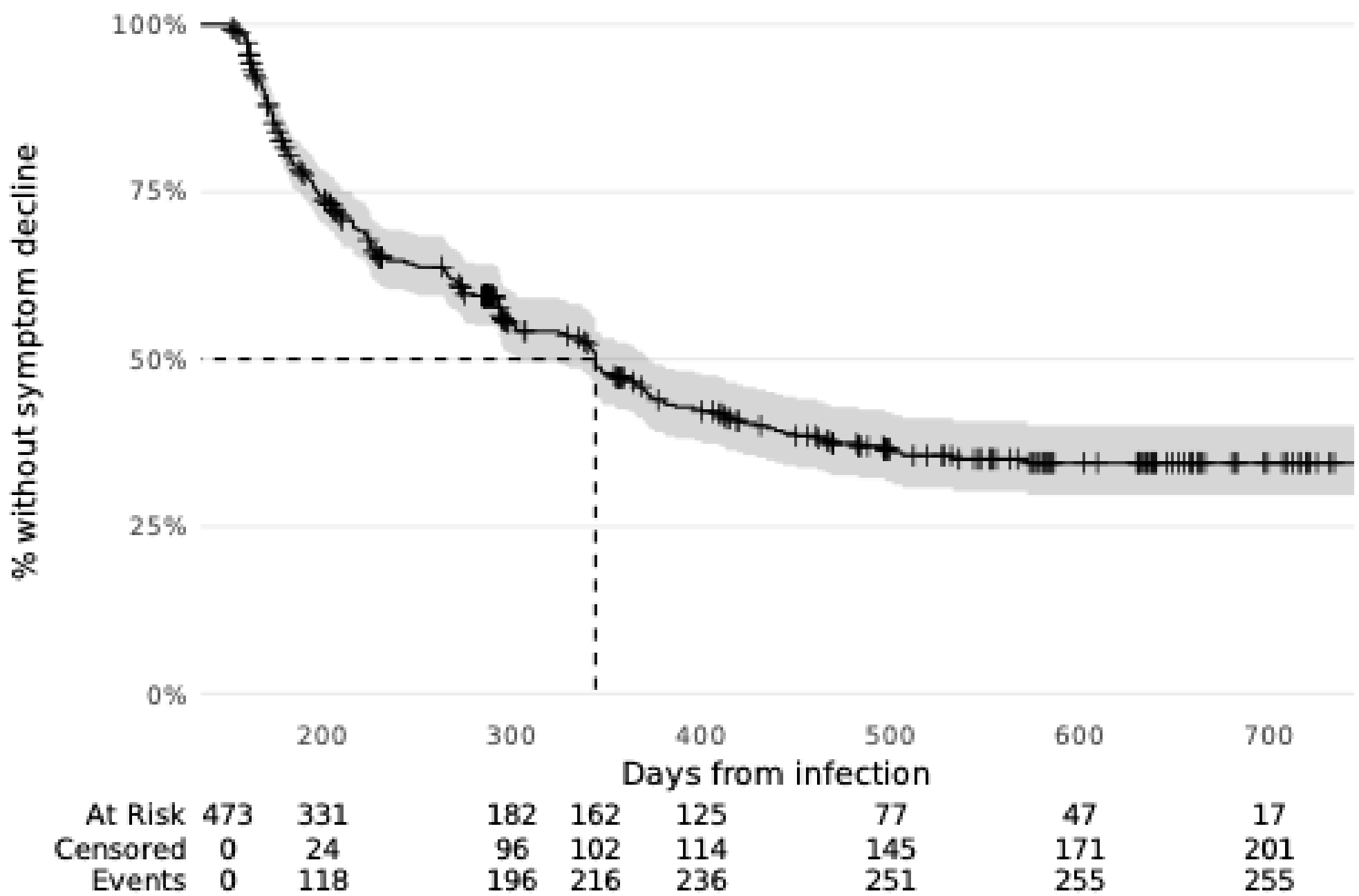

Figure S3 - Sensitivity analysis using a stricter definition of symptom recovery based on two consecutive questionnaires

	Number of events/at risk	HR (95% CI)	p-value
**Age group**			
Middle Adulthood (reference)	112/213	Reference	
Early Adulthood	19/30	2.34 (1.40–3.89)	0.0011
Late Adulthood	53/100	1.06 (0.76–1.49)	0.73
**Sex**			
Male (reference)	54/99	Reference	
Female	130/244	1.24 (0.89–1.73)	0.21
**Body mass index**			
Healthy weight (reference)	62/120	Reference	
Overweight	78/147	0.94 (0.66–1.33)	0.73
Obesity	44/76	1.14 (0.76–1.71)	0.52
**Comorbidities**			
No (reference)	98/187	Reference	
One chronic disease	42/81	0.98 (0.68–1.41)	0.91
Multiple chronic diseases	44/75	1.21 (0.83–1.76)	0.31
**Smoking**			
No (reference)	166/307	Reference	
Yes	18/36	0.93 (0.57–1.53)	0.78
**Hospitalization**			
No (reference)	175/329	Reference	
Yes	<10/14	1.31 (0.64–2.68)	0.46
**Vaccination status**			
No (reference)	163/277	Reference	
Complete vaccination	21/66	0.85 (0.39–1.86)	0.68
**Number of symptoms**			
Per symptom (unit)	184/343	0.68 (0.59–0.78)	<0.0001

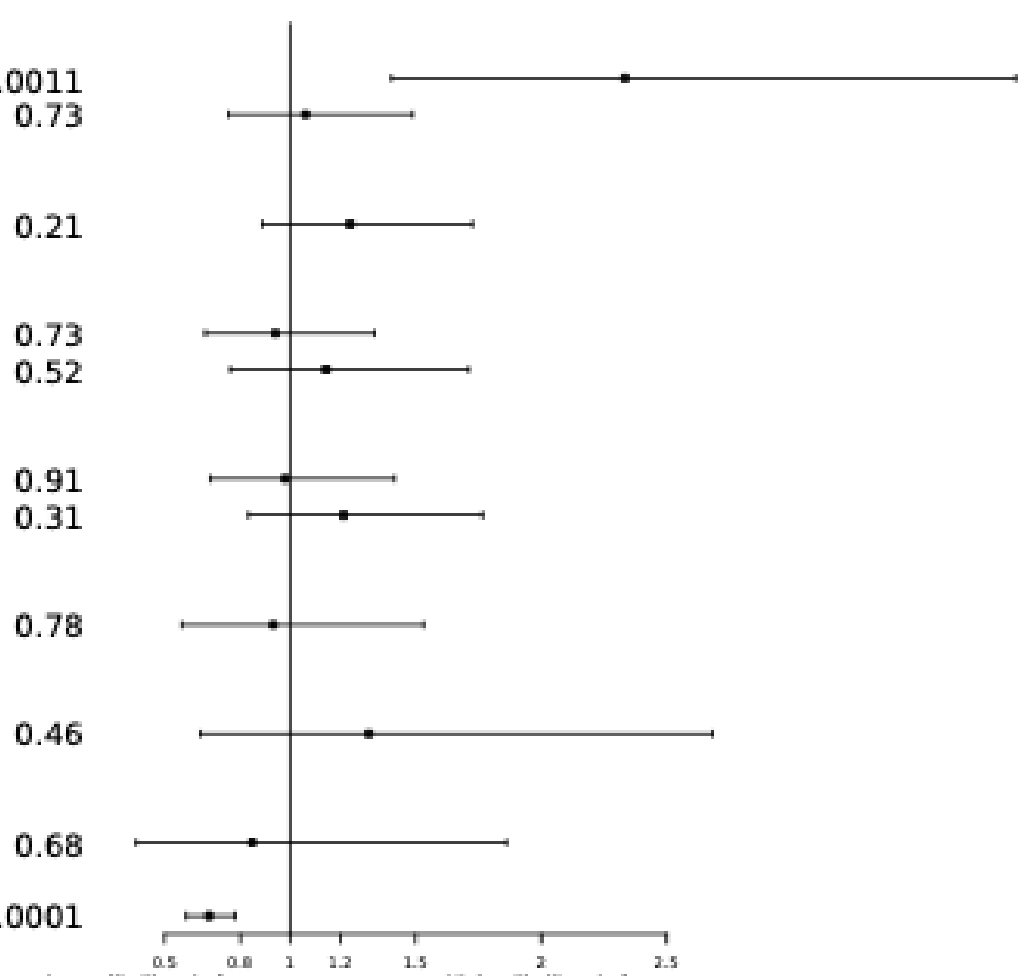

Figure S4 - Sensitivity analysis- Forest plot of hazard ratios from multivariable Cox proportional hazards models stratified by SARS-CoV-2 variant using the two-questionnaire definition of return to baseline symptom level. Category with less than 10 are reported as <10 to preserve participants anonymity.

	Number of events/at risk	HR (95% CI)	p-value
**Age group**			
Middle Adulthood (reference)	112/213	Reference	
Early Adulthood	19/30	2.55 (1.52–4.30)	0.00043
Late Adulthood	53/100	1.14 (0.81–1.62)	0.45
**Sex**			
Male (reference)	54/99	Reference	
Female	130/244	1.19 (0.84–1.67)	0.33
**Body mass index**			
Healthy weight (reference)	62/120	Reference	
Overweight	78/147	0.98 (0.69–1.41)	0.92
Obesity	44/76	1.24 (0.82–1.87)	0.3
**Comorbidities**			
No (reference)	98/187	Reference	
One chronic disease	42/81	0.89 (0.60–1.30)	0.54
Multiple chronic diseases	44/75	0.99 (0.67–1.46)	0.95
**Smoking**			
No (reference)	166/307	Reference	
Yes	18/36	1.07 (0.64–1.77)	0.8
**Hospitalization**			
No (reference)	175/329	Reference	
Yes	<10/14	0.99 (0.47–2.11)	0.99
**Vaccination status**			
No (reference)	163/277	Reference	
Complete vaccination	21/66	0.95 (0.42–2.15)	0.9
**Chest pain**			
No (reference)	168/308	Reference	
Yes	16/35	1.12 (0.64–1.95)	0.69
**Difficulties with breathing**			
No (reference)	169/305	Reference	
Yes	15/38	0.58 (0.31–1.06)	0.075
**Pain when breathing**			
No (reference)	179/324	Reference	
Yes	<10/19	0.47 (0.17–1.29)	0.14
**Painful muscles**			
No (reference)	118/224	Reference	
Yes	66/119	0.75 (0.53–1.06)	0.099
**Ageusia or anosmia**			
No (reference)	127/221	Reference	
Yes	57/122	0.42 (0.28–0.62)	<0.0001
**Tingling extremities**			
No (reference)	165/296	Reference	
Yes	19/47	0.79 (0.47–1.32)	0.37
**Lump in throat**			
No (reference)	174/310	Reference	
Yes	10/33	0.44 (0.23–0.86)	0.015
**Feeling hot and cold alternately**			
No (reference)	158/284	Reference	
Yes	26/59	0.81 (0.51–1.28)	0.37
**General tiredness**			
No (reference)	158/272	Reference	
Yes	26/71	0.50 (0.31–0.81)	0.0046
**Heavy arms or legs**			
No (reference)	151/257	Reference	
Yes	33/86	0.75 (0.48–1.17)	0.2
**Memory problems**			
No (reference)	181/321	Reference	
Yes	<10/22	0.16 (0.03–1.05)	0.056
**Concentration problems**			
No (reference)	180/321	Reference	
Yes	<10/22	1.47 (0.28–7.77)	0.65

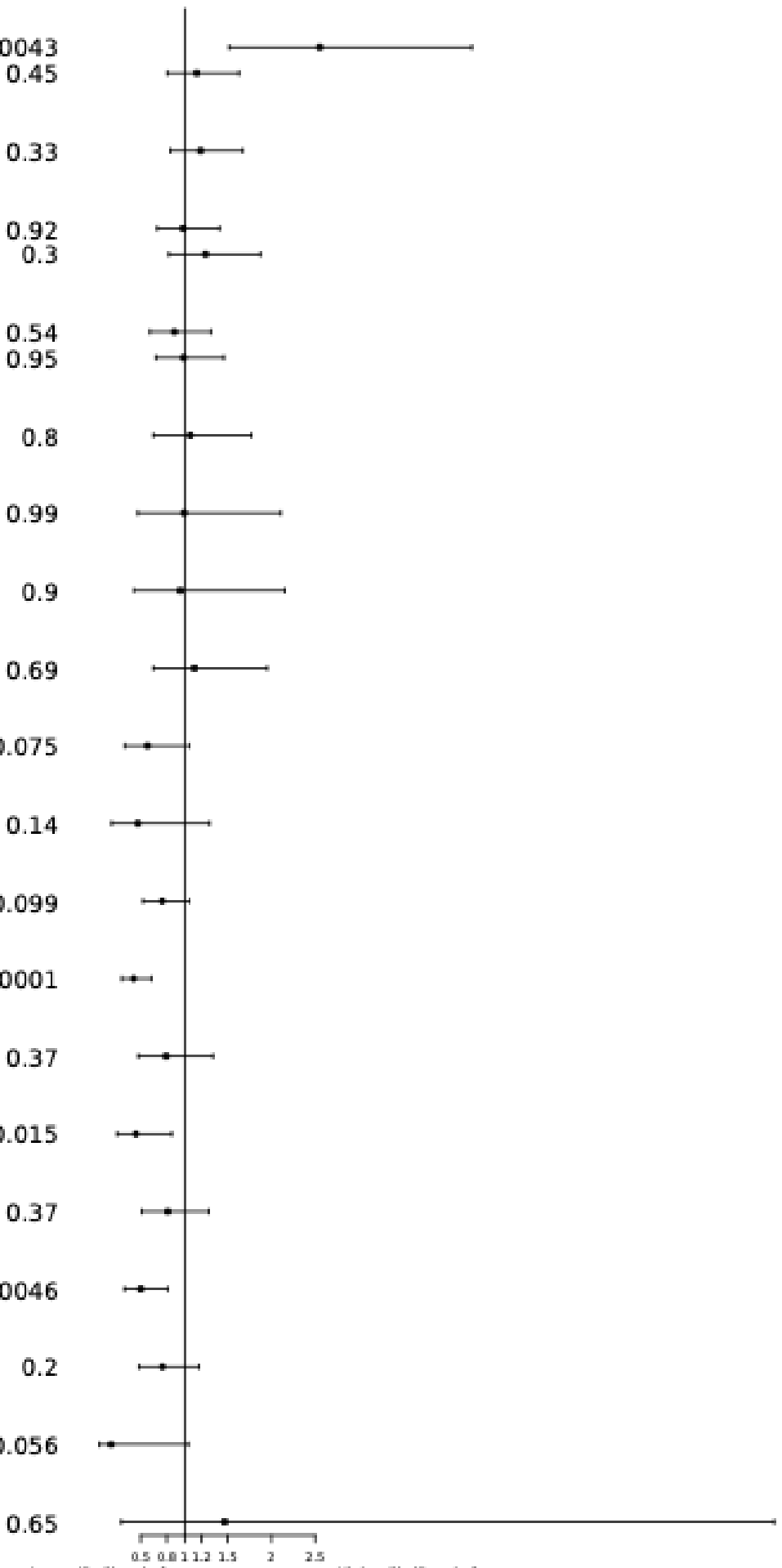

Figure S5 - Sensitivity analysis: - Forest plot of hazard ratios from multivariable Cox proportional hazards models for PCC symptoms stratified by variant considering return to baseline level for two questionnaires. Category with less than 10 are reported as <10 to preserve participants anonymity.

	Events/at risk/ missing	HR (95% CI)	p-value
**Age group**			
Middle Adulthood (reference)	334/491/0	Reference	
Early Adulthood	47/68/0	1.25 (0.91–1.72)	0.16
Late Adulthood	177/250/0	1.17 (0.97–1.42)	0.099
**Sex**			
Male (reference)	158/219/0	Reference	
Female	400/590/0	0.96 (0.79–1.16)	0.66
**Body mass index**			
Healthy weight (reference)	216/307/0	Reference	
Overweight	224/323/0	0.87 (0.72–1.05)	0.15
Obesity	118/179/0	1.05 (0.83–1.33)	0.7
**Comorbidities**			
No (reference)	283/404/60	Reference	
One chronic disease	110/171/60	1.03 (0.83–1.29)	0.76
Multiple chronic diseases	120/174/60	1.14 (0.91–1.42)	0.25
**Smoking**			
No (reference)	505/721/1	Reference	
Yes	52/87/1	0.93 (0.70–1.25)	0.65
**Hospitalization**			
No (reference)	528/766/11	Reference	
Yes	24/32/11	0.93 (0.61–1.44)	0.76
**Vaccination status**			
No (reference)	256/326/113	Reference	
Complete vaccination	221/370/113	1.14 (0.77–1.70)	0.5
**Number of symptoms**			
Per symptom (unit)	558/809/0	0.69 (0.64–0.75)	<0.0001

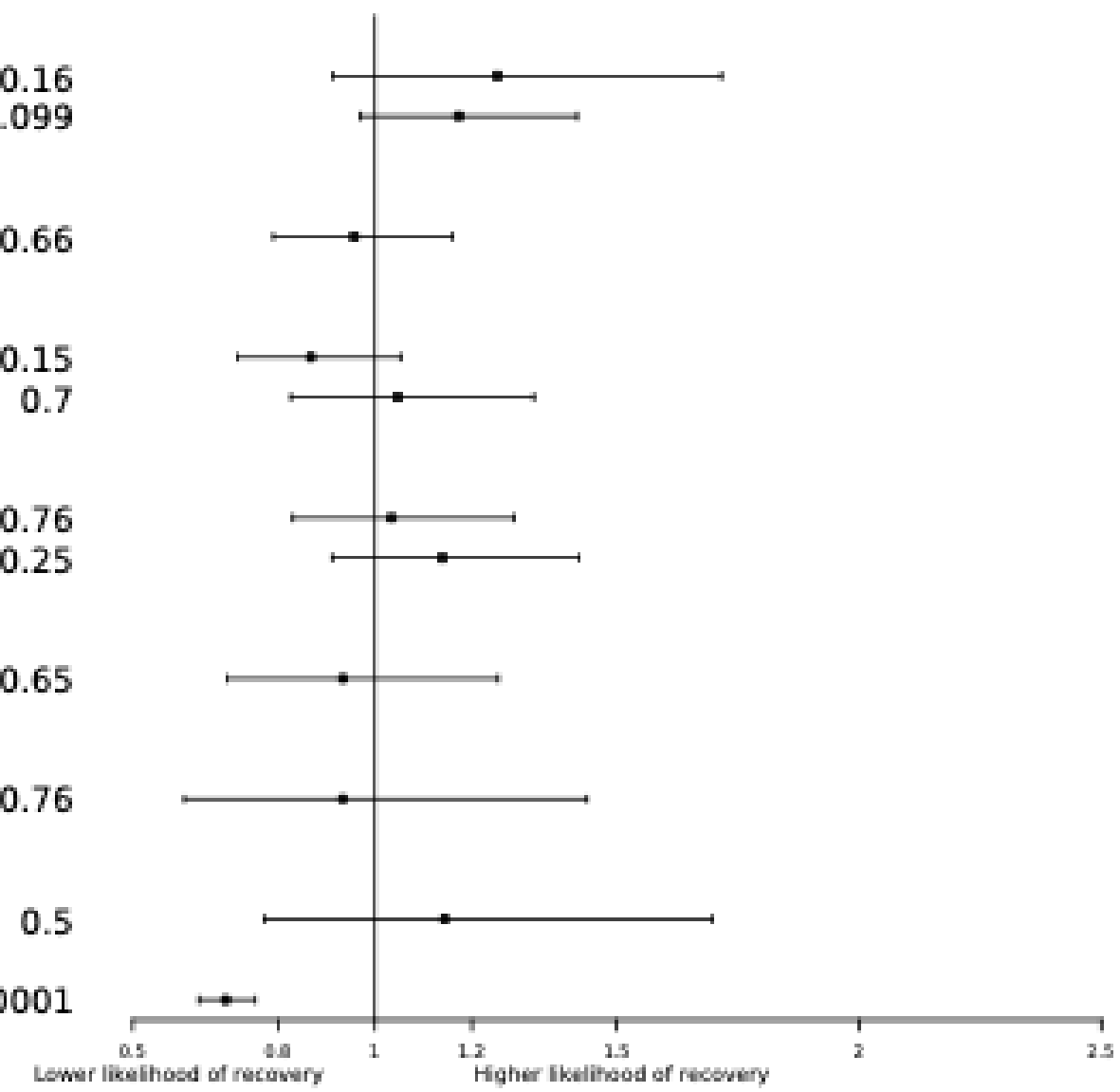

Figure S6 - Forest plot of hazard ratios from multivariable Cox proportional hazards models stratified by SARS-CoV-2 variant following multiple imputation.

	Events/at risk/ missing	HR (95% CI)	p-value
**Age group**			
Middle Adulthood (reference)	334/491/0	Reference	
Early Adulthood	47/68/0	1.31 (0.95–1.80)	0.099
Late Adulthood	177/250/0	1.24 (1.02–1.50)	0.034
**Sex**			
Male (reference)	158/219/0	Reference	
Female	400/590/0	0.88 (0.72–1.07)	0.21
**Body mass index**			
Healthy weight (reference)	216/307/0	Reference	
Overweight	224/323/0	0.86 (0.71–1.04)	0.13
Obesity	118/179/0	1.03 (0.81–1.31)	0.79
**Comorbidities**			
No (reference)	283/404/60	Reference	
One chronic disease	110/171/60	1.01 (0.81–1.27)	0.92
Multiple chronic diseases	120/174/60	1.08 (0.86–1.36)	0.5
**Smoking**			
No (reference)	505/721/1	Reference	
Yes	52/87/1	1.03 (0.76–1.39)	0.85
**Hospitalization**			
No (reference)	528/766/11	Reference	
Yes	24/32/11	0.78 (0.51–1.21)	0.27
**Vaccination status**			
No (reference)	256/326/113	Reference	
Complete vaccination	221/370/113	1.07 (0.72–1.59)	0.75
**Chest pain**			
No (reference)	522/747/0	Reference	
Yes	36/62/0	0.98 (0.69–1.41)	0.93
**Difficulties with breathing**			
No (reference)	516/732/0	Reference	
Yes	42/77/0	0.59 (0.41–0.83)	0.0029
**Pain when breathing**			
No (reference)	546/777/0	Reference	
Yes	12/32/0	0.67 (0.35–1.28)	0.22
**Painful muscles**			
No (reference)	360/507/0	Reference	
Yes	198/302/0	0.67 (0.55–0.82)	<0.0001
**Ageusia or anosmia**			
No (reference)	433/611/0	Reference	
Yes	125/198/0	0.37 (0.29–0.48)	<0.0001
**Tingling extremities**			
No (reference)	500/687/0	Reference	
Yes	58/122/0	0.61 (0.45–0.81)	0.0007
**Lump in throat**			
No (reference)	522/740/0	Reference	
Yes	36/69/0	0.62 (0.44–0.88)	0.0077
**Feeling hot and cold alternately**			
No (reference)	454/648/0	Reference	
Yes	104/161/0	0.85 (0.67–1.08)	0.19
**General tiredness**			
No (reference)	473/633/0	Reference	
Yes	85/176/0	0.57 (0.44–0.74)	<0.0001
**Heavy arms or legs**			
No (reference)	449/611/0	Reference	
Yes	109/198/0	0.84 (0.66–1.07)	0.16
**Memory problems**			
No (reference)	535/732/0	Reference	
Yes	23/77/0	0.41 (0.25–0.67)	0.00048
**Concentration problems**			
No (reference)	535/735/0	Reference	
Yes	23/74/0	0.64 (0.39–1.06)	0.081

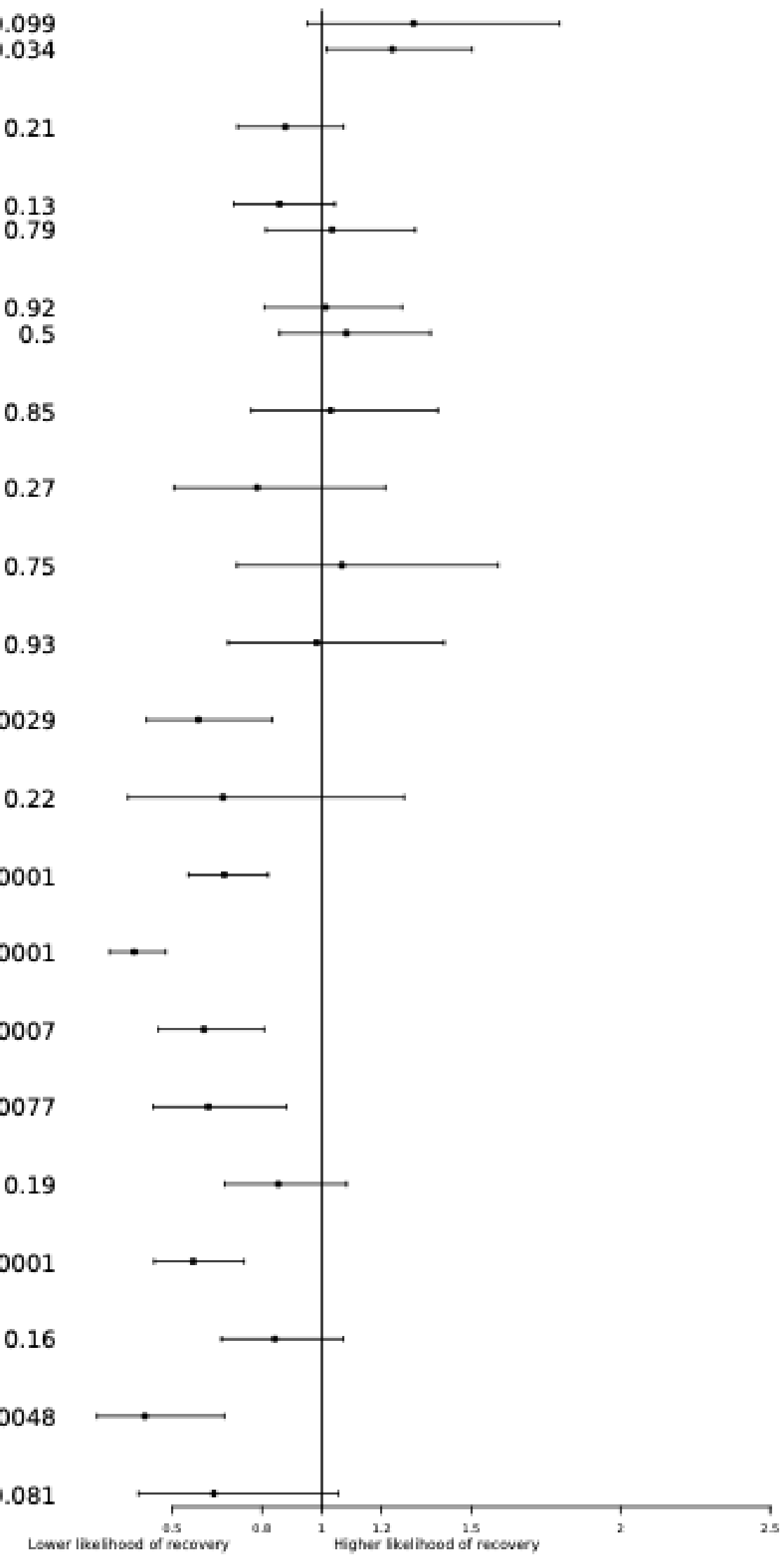

Figure S7 - Forest plot of hazard ratios from multivariable Cox proportional hazards models including symptoms stratified by SARS-CoV-2 variant following multiple imputation.

	Primary complete-case model	Multiple imputation (covariates)	IPCW for informative censoring	γ = -0.5	γ = -1	γ = 0.5	γ = 1
Age group							
Middle Adulthood (reference)	Reference	Reference	Reference	Reference	Reference	Reference	Reference
Early Adulthood	1.52 (1.06–2.18)<br>p=0.024	1.25 (0.91–1.72)<br>p=0.16	1.17 (0.77–1.78)<br>p=0.47	1.22 (0.89–1.69)<br>p=0.22	1.26 (0.92–1.72)<br>p=0.15	1.25 (0.92–1.70)<br>p=0.15	1.25 (0.93–1.67)<br>p=0.14
Late Adulthood	1.15 (0.92–1.42)<br>p=0.22	1.17 (0.97–1.42)<br>p=0.099	1.10 (0.88–1.37)<br>p=0.39	1.15 (0.95–1.39)<br>p=0.15	1.18 (0.98–1.41)<br>p=0.089	1.18 (0.99–1.42)<br>p=0.071	1.19 (1.00–1.42)<br>p=0.052
Sex							
Male (reference)	Reference	Reference	Reference	Reference	Reference	Reference	Reference
Female	1.08 (0.86–1.34)<br>p=0.51	0.96 (0.79–1.16)<br>p=0.66	1.03 (0.82–1.30)<br>p=0.78	0.97 (0.80–1.19)<br>p=0.79	0.96 (0.79–1.16)<br>p=0.68	0.96 (0.79–1.16)<br>p=0.65	0.96 (0.80–1.14)<br>p=0.62
Body mass index							
Healthy weight (reference)	Reference	Reference	Reference	Reference	Reference	Reference	Reference
Overweight	0.88 (0.70–1.09)<br>p=0.24	0.87 (0.72–1.05)<br>p=0.15	0.92 (0.74–1.14)<br>p=0.44	0.89 (0.73–1.08)<br>p=0.23	0.86 (0.71–1.05)<br>p=0.14	0.86 (0.71–1.04)<br>p=0.13	0.86 (0.72–1.03)<br>p=0.097
Obesity	1.06 (0.81–1.39)<br>p=0.66	1.05 (0.83–1.33)<br>p=0.7	0.98 (0.76–1.26)<br>p=0.87	1.03 (0.81–1.31)<br>p=0.79	1.05 (0.83–1.33)<br>p=0.69	1.07 (0.85–1.34)<br>p=0.57	1.07 (0.86–1.33)<br>p=0.54
Comorbidities							
No (reference)	Reference	Reference	Reference	Reference	Reference	Reference	Reference
One chronic disease	0.93 (0.73–1.18)<br>p=0.56	1.03 (0.83–1.29)<br>p=0.76	0.95 (0.73–1.25)<br>p=0.72	1.03 (0.82–1.29)<br>p=0.8	1.02 (0.82–1.28)<br>p=0.83	1.04 (0.84–1.28)<br>p=0.72	1.04 (0.85–1.28)<br>p=0.69
Multiple chronic diseases	1.13 (0.89–1.44)<br>p=0.32	1.14 (0.91–1.42)<br>p=0.25	1.10 (0.86–1.41)<br>p=0.45	1.16 (0.92–1.45)<br>p=0.2	1.13 (0.91–1.41)<br>p=0.26	1.13 (0.91–1.39)<br>p=0.27	1.11 (0.90–1.36)<br>p=0.34
Smoking							
No (reference)	Reference	Reference	Reference	Reference	Reference	Reference	Reference
Yes	0.86 (0.61–1.20)<br>p=0.37	0.93 (0.70–1.25)<br>p=0.65	0.77 (0.50–1.20)<br>p=0.25	0.90 (0.67–1.21)<br>p=0.49	0.93 (0.69–1.25)<br>p=0.63	0.96 (0.72–1.28)<br>p=0.77	0.99 (0.75–1.30)<br>p=0.94
Hospitalization							
No (reference)	Reference	Reference	Reference	Reference	Reference	Reference	Reference
Yes	1.24 (0.68–2.28)<br>p=0.49	0.93 (0.61–1.44)<br>p=0.76	1.02 (0.64–1.64)<br>p=0.92	0.95 (0.62–1.47)<br>p=0.83	0.93 (0.61–1.43)<br>p=0.75	0.92 (0.61–1.41)<br>p=0.71	0.91 (0.60–1.38)<br>p=0.66
Vaccination status							
No (reference)	Reference	Reference	Reference	Reference	Reference	Reference	Reference
Complete vaccination	1.10 (0.71–1.69)<br>p=0.68	1.14 (0.77–1.70)<br>p=0.5	1.23 (0.79–1.93)<br>p=0.36	1.07 (0.71–1.60)<br>p=0.76	1.13 (0.77–1.68)<br>p=0.53	1.18 (0.81–1.73)<br>p=0.39	1.21 (0.85–1.74)<br>p=0.29
Number of symptoms							
Per symptom (unit)	0.69 (0.63–0.76)<br>p<0.0001	0.69 (0.64–0.75)<br>p<0.0001	0.64 (0.57–0.71)<br>p<0.0001	0.68 (0.62–0.74)<br>p<0.0001	0.70 (0.64–0.75)<br>p<0.0001	0.71 (0.65–0.77)<br>p<0.0001	0.73 (0.68–0.79)<br>p<0.0001

Figure S8 - Sensitivity analyses of adjusted Cox proportional hazards models for recovery. Hazard ratios (HRs) with 95% confidence intervals are presented for the primary complete-case model, multiple imputation, IPCW after imputation for informative censoring, and gamma sensitivity analyses in the imputed dataset. HRs <1 indicate lower recovery likelihood and HRs >1 indicate higher recovery likelihood.

	Primary complete-case model	Multiple imputation (covariates)	IPCW for informative censoring	γ = -0.5	γ = -1	γ = 0.5	γ = 1
Age group							
Middle Adulthood (reference)	Reference	Reference	Reference	Reference	Reference	Reference	Reference
Early Adulthood	1.62 (1.13–2.34)<br>p=0.0092	1.31 (0.95–1.80)<br>p=0.099	1.23 (0.83–1.83)<br>p=0.29	1.29 (0.93–1.78)<br>p=0.13	1.30 (0.95–1.78)<br>p=0.11	1.30 (0.95–1.76)<br>p=0.099	1.29 (0.96–1.74)<br>p=0.097
Late Adulthood	1.24 (0.99–1.55)<br>p=0.061	1.24 (1.02–1.50)<br>p=0.034	1.19 (0.95–1.48)<br>p=0.13	1.22 (1.01–1.49)<br>p=0.044	1.24 (1.02–1.50)<br>p=0.033	1.24 (1.02–1.49)<br>p=0.028	1.24 (1.03–1.48)<br>p=0.022
Sex							
Male (reference)	Reference	Reference	Reference	Reference	Reference	Reference	Reference
Female	1.00 (0.79–1.25)<br>p=0.98	0.88 (0.72–1.07)<br>p=0.21	0.93 (0.74–1.18)<br>p=0.57	0.89 (0.72–1.09)<br>p=0.25	0.88 (0.72–1.08)<br>p=0.23	0.89 (0.73–1.08)<br>p=0.22	0.89 (0.74–1.08)<br>p=0.23
Body mass index							
Healthy weight (reference)	Reference	Reference	Reference	Reference	Reference	Reference	Reference
Overweight	0.88 (0.71–1.10)<br>p=0.26	0.86 (0.71–1.04)<br>p=0.13	0.90 (0.73–1.10)<br>p=0.31	0.87 (0.72–1.07)<br>p=0.18	0.86 (0.71–1.04)<br>p=0.13	0.86 (0.71–1.04)<br>p=0.11	0.86 (0.71–1.02)<br>p=0.09
Obesity	1.09 (0.83–1.44)<br>p=0.53	1.03 (0.81–1.31)<br>p=0.79	0.99 (0.77–1.27)<br>p=0.93	1.02 (0.80–1.30)<br>p=0.9	1.04 (0.82–1.32)<br>p=0.75	1.05 (0.83–1.33)<br>p=0.67	1.06 (0.85–1.33)<br>p=0.61
Comorbidities							
No (reference)	Reference	Reference	Reference	Reference	Reference	Reference	Reference
One chronic disease	0.91 (0.71–1.17)<br>p=0.46	1.01 (0.81–1.27)<br>p=0.92	0.94 (0.72–1.23)<br>p=0.67	1.01 (0.80–1.27)<br>p=0.94	1.01 (0.81–1.25)<br>p=0.96	1.01 (0.81–1.26)<br>p=0.92	1.01 (0.82–1.25)<br>p=0.91
Multiple chronic diseases	1.06 (0.82–1.36)<br>p=0.67	1.08 (0.86–1.36)<br>p=0.5	1.04 (0.81–1.34)<br>p=0.76	1.10 (0.87–1.39)<br>p=0.44	1.08 (0.85–1.35)<br>p=0.54	1.07 (0.85–1.33)<br>p=0.56	1.06 (0.85–1.31)<br>p=0.61
Smoking							
No (reference)	Reference	Reference	Reference	Reference	Reference	Reference	Reference
Yes	0.94 (0.67–1.32)<br>p=0.73	1.03 (0.76–1.39)<br>p=0.85	0.86 (0.58–1.28)<br>p=0.46	1.00 (0.73–1.35)<br>p=0.98	1.03 (0.76–1.39)<br>p=0.87	1.06 (0.80–1.41)<br>p=0.69	1.08 (0.82–1.42)<br>p=0.59
Hospitalization							
No (reference)	Reference	Reference	Reference	Reference	Reference	Reference	Reference
Yes	0.86 (0.46–1.61)<br>p=0.64	0.78 (0.51–1.21)<br>p=0.27	0.77 (0.47–1.26)<br>p=0.3	0.81 (0.53–1.25)<br>p=0.34	0.78 (0.50–1.21)<br>p=0.27	0.77 (0.50–1.19)<br>p=0.24	0.77 (0.50–1.17)<br>p=0.21
Vaccination status							
No (reference)	Reference	Reference	Reference	Reference	Reference	Reference	Reference
Complete vaccination	1.00 (0.65–1.56)<br>p=0.99	1.07 (0.72–1.59)<br>p=0.75	1.07 (0.69–1.66)<br>p=0.76	1.01 (0.68–1.51)<br>p=0.96	1.07 (0.72–1.59)<br>p=0.73	1.10 (0.75–1.62)<br>p=0.63	1.13 (0.78–1.63)<br>p=0.53
Chest pain							
No (reference)	Reference	Reference	Reference	Reference	Reference	Reference	Reference
Yes	0.99 (0.66–1.50)<br>p=0.97	0.98 (0.69–1.41)<br>p=0.93	0.76 (0.47–1.23)<br>p=0.27	1.04 (0.72–1.48)<br>p=0.85	1.00 (0.70–1.43)<br>p=0.99	0.98 (0.69–1.39)<br>p=0.92	0.97 (0.69–1.37)<br>p=0.88
Difficulties with breathing							
No (reference)	Reference	Reference	Reference	Reference	Reference	Reference	Reference
Yes	0.55 (0.37–0.81)<br>p=0.0029	0.59 (0.41–0.83)<br>p=0.0029	0.49 (0.31–0.78)<br>p=0.0027	0.57 (0.40–0.82)<br>p=0.0024	0.59 (0.41–0.84)<br>p=0.0033	0.60 (0.43–0.85)<br>p=0.0041	0.63 (0.46–0.88)<br>p=0.0062
Pain when breathing							
No (reference)	Reference	Reference	Reference	Reference	Reference	Reference	Reference
Yes	0.57 (0.28–1.16)<br>p=0.12	0.67 (0.35–1.28)<br>p=0.22	0.67 (0.24–1.90)<br>p=0.46	0.71 (0.37–1.35)<br>p=0.29	0.67 (0.35–1.27)<br>p=0.22	0.65 (0.35–1.23)<br>p=0.19	0.65 (0.35–1.22)<br>p=0.18
Painful muscles							
No (reference)	Reference	Reference	Reference	Reference	Reference	Reference	Reference
Yes	0.68 (0.54–0.85)<br>p=0.00056	0.67 (0.55–0.82)<br>p<0.0001	0.63 (0.50–0.78)<br>p<0.0001	0.68 (0.55–0.82)<br>p=0.00012	0.68 (0.56–0.82)<br>p<0.0001	0.68 (0.56–0.83)<br>p=0.00012	0.70 (0.59–0.84)<br>p=0.00013
Ageusia or anosmia							
No (reference)	Reference	Reference	Reference	Reference	Reference	Reference	Reference
Yes	0.38 (0.29–0.51)<br>p<0.0001	0.37 (0.29–0.48)<br>p<0.0001	0.31 (0.22–0.43)<br>p<0.0001	0.37 (0.29–0.47)<br>p<0.0001	0.37 (0.29–0.48)<br>p<0.0001	0.38 (0.30–0.49)<br>p<0.0001	0.40 (0.32–0.51)<br>p<0.0001
Tingling extremities							
No (reference)	Reference	Reference	Reference	Reference	Reference	Reference	Reference
Yes	0.63 (0.45–0.86)<br>p=0.0042	0.61 (0.45–0.81)<br>p=0.0007	0.61 (0.43–0.87)<br>p=0.0065	0.58 (0.43–0.78)<br>p=0.00029	0.61 (0.45–0.81)<br>p=0.00065	0.63 (0.48–0.83)<br>p=0.0013	0.67 (0.51–0.87)<br>p=0.0026
Lump in throat							
No (reference)	Reference	Reference	Reference	Reference	Reference	Reference	Reference
Yes	0.60 (0.40–0.91)<br>p=0.015	0.62 (0.44–0.88)<br>p=0.0077	0.63 (0.41–0.99)<br>p=0.043	0.57 (0.40–0.81)<br>p=0.002	0.62 (0.44–0.88)<br>p=0.0084	0.66 (0.48–0.93)<br>p=0.016	0.70 (0.51–0.96)<br>p=0.027
Feeling hot and cold alternately							
No (reference)	Reference	Reference	Reference	Reference	Reference	Reference	Reference
Yes	0.95 (0.73–1.24)<br>p=0.72	0.85 (0.67–1.08)<br>p=0.19	0.86 (0.65–1.13)<br>p=0.28	0.87 (0.68–1.11)<br>p=0.27	0.85 (0.67–1.08)<br>p=0.18	0.84 (0.67–1.06)<br>p=0.14	0.84 (0.67–1.04)<br>p=0.11
General tiredness							
No (reference)	Reference	Reference	Reference	Reference	Reference	Reference	Reference
Yes	0.60 (0.44–0.81)<br>p=0.00077	0.57 (0.44–0.74)<br>p<0.0001	0.48 (0.35–0.65)<br>p<0.0001	0.55 (0.42–0.71)<br>p<0.0001	0.58 (0.44–0.75)<br>p<0.0001	0.60 (0.46–0.77)<br>p<0.0001	0.63 (0.49–0.80)<br>p=0.00019
Heavy arms or legs							
No (reference)	Reference	Reference	Reference	Reference	Reference	Reference	Reference
Yes	0.82 (0.62–1.07)<br>p=0.15	0.84 (0.66–1.07)<br>p=0.16	0.98 (0.74–1.28)<br>p=0.86	0.83 (0.65–1.05)<br>p=0.12	0.84 (0.66–1.06)<br>p=0.14	0.85 (0.68–1.07)<br>p=0.16	0.87 (0.70–1.08)<br>p=0.21
Memory problems							
No (reference)	Reference	Reference	Reference	Reference	Reference	Reference	Reference
Yes	0.48 (0.28–0.83)<br>p=0.0088	0.41 (0.25–0.67)<br>p=0.00048	0.35 (0.21–0.58)<br>p<0.0001	0.40 (0.24–0.66)<br>p=0.00046	0.41 (0.25–0.67)<br>p=0.00041	0.43 (0.27–0.69)<br>p=0.00049	0.46 (0.29–0.72)<br>p=0.00069
Concentration problems							
No (reference)	Reference	Reference	Reference	Reference	Reference	Reference	Reference
Yes	0.60 (0.35–1.02)<br>p=0.06	0.64 (0.39–1.06)<br>p=0.081	0.50 (0.30–0.82)<br>p=0.0065	0.59 (0.35–1.00)<br>p=0.049	0.63 (0.39–1.03)<br>p=0.064	0.66 (0.42–1.05)<br>p=0.083	0.69 (0.45–1.07)<br>p=0.1

Figure S9 - Sensitivity analyses of adjusted Cox proportional hazards models including symptoms for recovery. Hazard ratios (HRs) with 95% confidence intervals are presented for the primary complete-case model, multiple imputation, IPCW for informative censoring, and gamma sensitivity analyses. HRs <1 indicate lower recovery likelihood and HRs >1 indicate higher recovery likelihood.

Table S2 Questionnaire participation and number of observations contributing to baseline and PCC symptom estimation.

Measure	Number of participants	Median questionnaires per participant (IQR)	Median Observation range in days (IQR) (time between first and last questionnaire in observation window)
Pre-infection baseline estimation	12412	19 (11-24)	559 (286-634)
PCC diagnostic window (90-150 days)	6102	1 (1-2)	0 (0-26) Most participants only have a singular time point

Lifelines Corona Research Initiative:

H. Marike Boezen[1], Jochen O. Mierau[2,3,4], H. Lude Franke[5] , Jackie Dekens[5,6], Patrick Deelen[5] , Pauline Lanting[5] , Judith M. Vonk[1] , Ilja Nolte[1] , Anil P.S. Ori[5,7], Annique Claringbould[5] , Floranne Boulogne[5] , Marjolein X.L. Dijkema[5] , Henry H. Wiersma[5] , Robert Warmerdam[5] , Soesma A. Jankipersadsing[5] , Irene van Blokland[5,8], Cisca Wijmenga[5] , Geertruida H. de Bock[1] , Judith G.M. Rosmalen[7,9].

1 Department of Epidemiology, University of Groningen, University Medical Center Groningen, Groningen, The Netherlands
2 Department of Economics, Econometrics & Finance, Faculty of Economics and Business, University of Groningen, Groningen, The Netherlands
3 Team Strategy &External Relations, University of Groningen, University Medical Center Groningen, the Netherlands
4 Lifelines Cohort Study and Biobank, Groningen, The Netherlands
5 Department of Genetics, University of Groningen, University Medical Center Groningen, Groningen, The Netherlands
6 Center of Development and Innovation, University of Groningen, University Medical Center Groningen, Groningen, The Netherlands
7 Department of Psychiatry, University of Groningen, University Medical Center Groningen, Groningen, The Netherlands
8 Department of Cardiology, University of Groningen, University Medical Center Groningen, Groningen, The Netherlands
9 Department of Internal Medicine, University of Groningen, University Medical Center Groningen, Groningen, The Netherlands
